# Production of Paralytic Shellfish Toxins (PSTs) in Toxic *Alexandrium catenella* is Intertwined with Photosynthesis and Energy Production

**DOI:** 10.3390/toxins12080477

**Published:** 2020-07-27

**Authors:** Sirius Pui-kam Tse, Fred Wang-fat Lee, Daniel Yun-lam Mak, Hang-kin Kong, Kenrick Kai-yuen Chan, Pak-yeung Lo, Samuel Chun-lap Lo

**Affiliations:** 1 The Department of Applied Biology and Chemical Technology, The Hong Kong Polytechnic University, Hong Kong; pktse@polyu.edu.hk (S.P.-k.T.); lampiano@hotmail.com (D.Y.-l.M.); hang-kin.kong@polyu.edu.hk (H.-k.K.); kai-yuen.ky.chan@connect.polyu.hk (K.K.-y.C.); pat_lo22@hotmail.com (P.-y.L.); 2 Department of Science, School of Science and Technology, The Open University of Hong Kong, Hong Kong; wflee@ouhk.edu.hk

**Keywords:** *Alexandrium catenella*, paralytic shellfish toxins, PSTs, light availability, phosphate limitation, proteomics

## Abstract

To investigate the mechanism for the production of paralytic shellfish toxins (PST) in toxic dinoflagellates, with a 2D-gel based approach, we had made two sets of proteomic comparisons: (a) between a toxic *Alexandrium catenella* (AC-T) and a phylogenetically closely related non-toxic strain (AC-N), (b) between toxic AC-T grown in a medium with 10% normal amount of phosphate (AC-T-10%P) known to induce higher toxicity and AC-T grown in normal medium. We found that photosynthesis and energy production related proteins were up-regulated in AC-T when compared to AC-N. However, the same group of proteins was down-regulated in AC-T-10%P when compared to normal AC-T. Examining the relationship of photosynthesis and toxin content of AC-T upon continuous photoperiod experiment revealed that while growth and associated toxin content increased after 8 days of continuous light, toxin content maintained constant when cells were shifted from continuous light to continuous dark for 3 days. This emphasized the cruciality of light availability on toxin biosynthesis in AC-T, while another light-independent mechanism may be responsible for higher toxicity in AC-T-10%P compared to normal AC-T. Taken all together, it is believed that the interplay between “illumination”, “photosynthesis”, “phosphate availability”, and “toxin production” is much more complicated than what we had previously anticipated.

## 1. Introduction

Paralytic shellfish poisoning (PSP) is the intoxication with paralytic shellfish toxins (PST) caused by the consumption of contaminated shellfish. PST poses a serious threat to public health because of its potential high mortality as well as rapid onset [1,2]. The occurrence of harmful algal blooms (HABs) induced by PST-producing microalgae could bring forth enormous negative economic impact as it may cause prolonged closure of molluscan shellfish harvesting areas and fishing grounds.

*Alexandrium*, *Gymnodinium,* and *Pyrodinium* are the three genera of marine dinoflagellates that are known to produce PST [3]. Phycological studies had found that there are variations of toxin content and profile among different species as well as strains of the dinoflagellates [4,5,6]. Other studies reported that the extent of PST production of these organisms could be influenced by certain physical or nutritional factors, such as the increase in toxicity of *Alexandrium sp.* under phosphate limitation [7,8,9] or under increasing ammonium concentration as primary nitrogen sources [10,11,12]. Van de Waal et al. [13] extracted 100 datasets from previous studies investigating the effects of N and/or P sources limitation on marine toxin production by phytoplankton and eventually, predicted by ecological stoichiometry, that PST as a nitrogen-rich toxin family would generally increase upon P-limitation but decrease upon N-limitation. On the basis of these facts and in order to understand the mechanism of PST production, researchers compared the differences in genetic makeup and gene expression levels [14,15,16], as well as physiological properties among toxic and non-toxic algal strains in determining the differences in abilities to produce toxins. For example, the production of PSTs under the G1 phase was suggested in PST-producing *A. fundyense* [17] and was further validated by the G1 phase inhibitor experiment in another PST-producing species *A. tamarense* [18,19]. Nevertheless, only limited information on the PST production mechanism was gathered.

*Sxt* clusters are a group of 26 genes reported to be crucial for PST biosynthesis in several cyanobacteria which also produce PST [14,15]. When the same knowledge was applied to dinoflagellates, several *sxt* gene clusters were found in the nuclear transcripts of PST-producing *A. fundyense* and *A. minutum* [16]. *SxtA*, the predicted starting gene of PST-producing cyanobacteria, was further investigated and it was reported that the gene structure of *sxtA* in dinoflagellates process certain differences to that of cyanobacteria, including that it has spliced-leader sequence, it is monocistronic in nature, has high GC content, occurs in multiple copies, and has polyA-tails of eukaryote [16]. In addition to the differences in gene structure, several additional points are noteworthy. Firstly, not all *sxt* genes in the cluster were found in the transcript library of both *A. fundyense* and *A. minutum* [16]. Secondly, there are two kinds of *sxtA* transcripts which differed by the numbers of catalytic domains present in *A. fundyense* [16] and in *A. minutum* [20], and this is likely to be similar in other *Alexandrium* species that contain the longer form of the *sxtA*4 transcript, such as *A. catenella*, *A. tamarense*, and *G. catenatum* [16]. Thirdly, the possibility of horizontal gene transfer of *sxt* genes in PST-producing cyanobacteria was suggested, indicating the difference in origins of some *sxt* genes in these organisms [15]. These findings implied that the biosynthetic mechanism of PST production in dinoflagellates is not identical to that in cyanobacteria. Furthermore, some non-toxic dinoflagellates, e.g., *A. tamarense*, also inherit *sxtA* gene domains [16]. Apart from the study of *sxtA* gene described above, there was also an attempt of adding 5-fluoro-2′-deoxyuridine (FUdR), a cytidine deaminase inhibitor, to inhibit the toxin production of *A. tamarense,* probably by inhibiting the activity of *sxtB*, which is believed to be an analog of cytidine deaminase [21]. However, as the exact gene products of *sxtB* were also yet to be identified, the exact pathway of toxin production is not clear as there is missing information about most of the corresponding proteins of *sxt* clusters in the species studied.

On another front, proteins are cellular workhorses that drive different metabolic pathways. Therefore, regulations of toxin production must be attributed to changes in expressions of related proteins. Hence, investigating differential protein expressions is reasonably one of the first steps to explore the mechanism of toxin synthesis. Such an approach with quantitative proteomics was reported in 2012 [22] and was refreshed by the same group in 2015 [23] and 2018 [19]. By comparing protein expression profiles of PST-producing *A. catenella* (AC) and its toxicity-lost mutant in both 2-dimensional gel electrophoresis [22] and iTRAQ-based shotgun proteomic basis [19,23], they reported the up-regulation of light-harvesting related proteins and methionine biosynthesis in the toxic strain. Further, seven *sxt* gene-related suspects were identified but none of them were differentially expressed in-between the toxic and non-toxic counterparts [19,23]. There are several inadequacies in these studies. Firstly, a large number of proteins reported in these studies were enzymes of known biological pathways such as light-harvesting, chlorophyll biosynthesis and photosynthesis, and the relationship between their expression levels and toxin contents were not elaborated. Secondly, the toxicity-lost mutant strain was found to have a different growth pattern when compared to its wild type, indicating other physiological differences in addition to the loss of toxicity. Lastly, no synchronization of growth phases was applied prior to some of the experiments [22,23], such that proteomes of the two strains might represent snapshots of mixtures of different cell stages, which introduced additional variations into the comparison. A similar case was reported which included the use of a non-toxic *A. lusitanicum* clone and it also had a different growth pattern when compared to its toxic counterparts [24]. The difference in growth patterns reflected that other metabolic pathways in addition to PST production were affected either by a substantial amount of mutation which might include genes of other essential pathways or as a metabolic unbalance. These uncertainties emphasize the need for a pair of *Alexandrium spp.* which are genetically closely related and with similar cellular physiology with the exception of toxigenicity for fair comparison purposes. After toxicity and physiological screening among our algal library, two *A. catenella* strains (which belong to group IV of the *A. tamarense* complex [25]) were found to have similar growth curves and one of them is PST-producing (AC-T) while the other is not (AC-N). Their close genetic proximity and physiological similarities were demonstrated by their similarities in growth curves, results of ITS, and rDNA sequences alignment as well as cell sizes. Hence, physiological differences between these two strains were thought to be minimal except for their toxin synthesizing abilities. Protein expression profiles of the two strains were compared using gel-based proteomic comparison. Identification of differentially expressed proteins was performed with MALDI-TOF-MS and LC-ESI-iontrap MS/MS analyses. Further, in order to eliminate the possible variations brought in the comparison due to differences in the genomes, another comparison was made to compare differential protein expressions in the same strain of toxic AC-T but under different environmental conditions. In *Alexandrium*, enhancement of toxin production under phosphate limitation was documented by several groups [7,8,9]. It was suggested that such an increase was due to the accumulation of produced toxins inside undivided cells [26]. Other types of quantification of toxin content including toxin amount per volume of cell or per protein amount had not been studied.

As elaborated, two proteomic comparisons were studied. Firstly, a comparison was made between a PST-producing *A. catenella* (AC-T) strain and another non-toxic *A. catenella* strain with 97% homology (AC-N). Secondly, to eliminate variations of the genomes (despite very high homology), AC-T cells were compared to AC-T cells grown in a 10% phosphate-limiting condition (AC-T-10%P). We already observed that AC-T-10%P cells are at least four times more toxic (in terms of toxin per cell and toxin per protein). We had found although photosynthesis and energy-related proteins were up-regulated in AC-T compared to AC-N, the same sets of proteins were down-regulated in more-toxic AC-T under phosphate limitation. The apparent opposite sets of results are interesting, but its interpretation is difficult. Nonetheless, to follow this up, we investigated if light and photosynthesis are the drivers for PST production and because AC-T-10%P cells did not survive well with prolonged cultivation, AC-T cells were subjected to a total 17-day long photoperiod experiment. Cells were grown in continuous light for 8 days (AC-T-L8) before changing to 3 days (AC-T-D3), 6 days (AC-T-D6), and 9 days of darkness (AC-T-D9) respectively. Our results on the first 14 days of the experiment showed that light and photosynthesis are primary drivers for PST production in AC-T cells. When AC-T cells were still surviving after 3 days of darkness, neither new toxin was produced nor secreted/excreted to the culture media.

## 2. Results

We isolated mono-clones of the toxic AC-T locally from the Silver Mine Bay of Hong Kong in March 2009. The non-toxic AC-N was cultivated from a single-cell isolate of an *Alexandrium* culture (CS-319) obtained from the Australian National Algae Culture Collection (ANACC). Morphological examination revealed consistent details with *Alexandrium catenella* (AC). Nonetheless, the molecular biology method was used to confirm the identities of these AC isolates. Another non-toxic dinoflagellate, *A. tamarense* (CCMP116), denoted as AT01, was obtain from the National Center of Marine Algae and Microbiota (NCMA).

### 2.1. DNA-Based Identification

**Figure 1:**
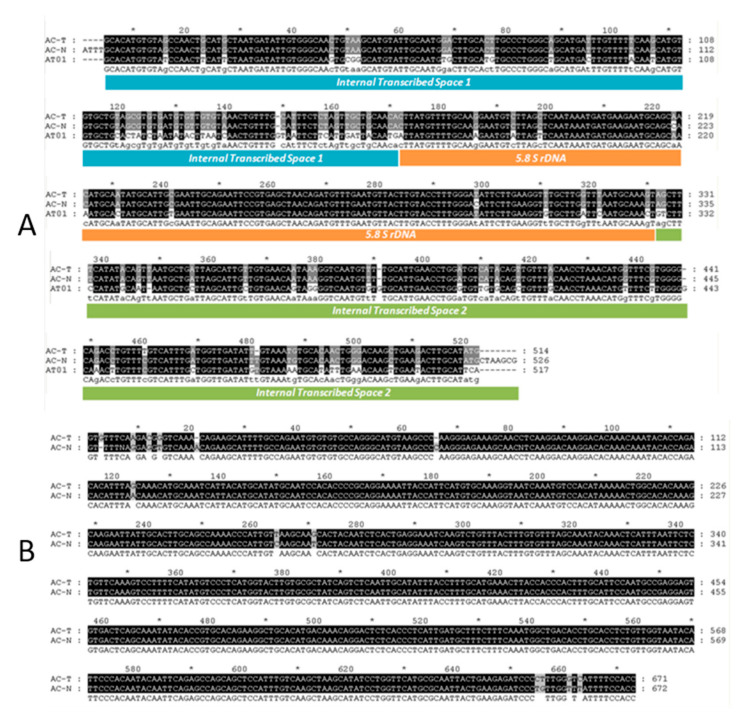
Alignments of (**A**) ITS1-5.8s-ITS2 sequences of AC-T and AC-N, together with the same sequence region of *A. tamarense* (AT01), and (**B**): partial 28S ribosomal sequences of AC-T and AC-N. There are 97% similarities between AC-T and AC-N that only a few base pair differences are found, whereas the similarities of the two *A. catenella* and *A. tamarense* (AT01) were 82%.

Alignments of the complete ITS1-5.8S-ITS2 sequences and partial 28S between AC-T and AC-N are shown in Figure 1. Both the ITS-5.8S-ITS2 (Figure 1A) and partial 28S (Figure 1B) alignments indicated that AC-T and AC-N shared a 97% homology. They were identified as *A. catenella* by their ITS1-5.8S-ITS2 sequences and were also suggested to belong to group IV of the *A. tamarense* complex according to the NCBI Genbank database [25,27]. Conversely, AC-T and AT01 shared 82% homology.

### 2.2. Growth Curves

**Figure 2:**
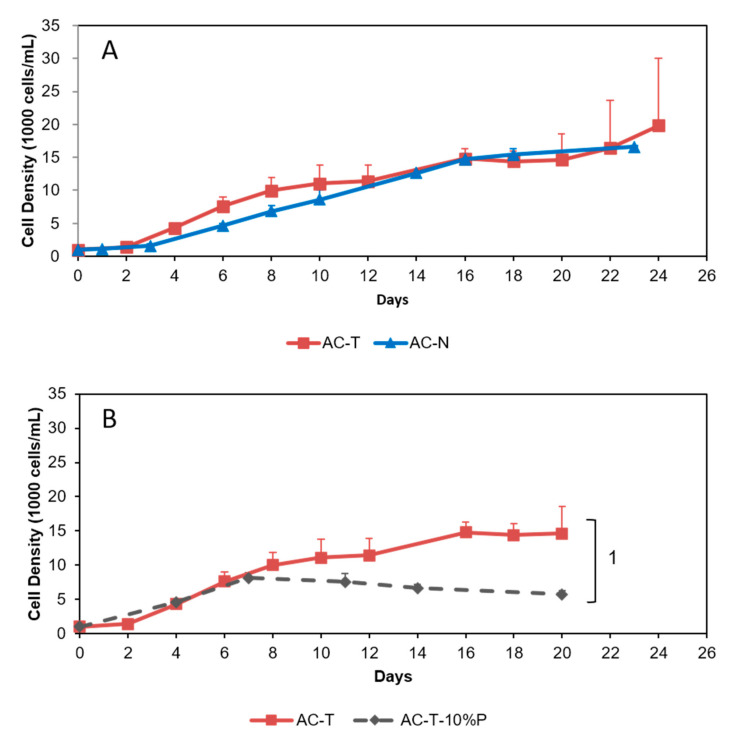
Growth curves of (**A**): from day 0 to day 24 of normal culture of AC-T and AC-N, and (**B**): from day 0 to day 20 of normal culture of AC-T and phosphate-limiting culture of AC-T (AC-T-10%P). Significant difference of cell density between AC-T and AC-T-10%P on day 20 (marked as 1) was verified using Student’s T-test with threshold *p* < 0.05.

Under normal culturing conditions, growth curves of AC-T and AC-N showed similar patterns (Figure 2). Specific growth rates of both AC-T and AC-N from day 2–20 were 0.12 day^−1^ (Figure 2A). The cell density of both cultures at the sampling time point (day 20) was around 14,000 to 16,000 cells/mL. Under phosphate limitation (10% PO_4_ of normal L1 medium), AC-T showed a different growth pattern with exponential growth stopped on day 7 (Figure 2B). Cell density was 8000 cells/mL at that time.

### 2.3. Cell Sizes of AC-N, AC-T, and AC-T with Phosphate Limitation

**Figure 3:**
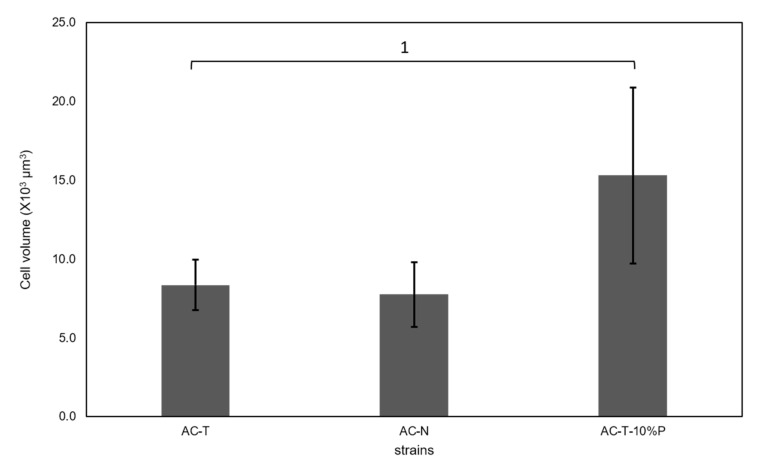
Cell volumes of AC-T, AC-N, and AC-T under 10% phosphate availability (AC-T-10%P) on day 20. A significant difference between cell volumes of AC-T and AC-T-10%P (marked as 1) was verified using Student’s T-test with threshold *p* < 0.05.

Mean cell volumes of these dinoflagellates including AC-T with normal provision of phosphate were estimated by light microscopy as previously reported [28]. Cells of AC-T and AC-N on day 20 had nearly constant mean cell volumes of (8.4 ± 1.6) × 10^3^ µm^3^ and (7.8 ± 2.1) × 10^3^ µm^3^ respectively. However, when put in L1 medium with only 10% phosphate provision, the cell size of AC-T increased drastically to (15.3 ± 5.6) × 10^3^ µm^3^ on day 20 (^1^: *p* = 0.014) (Figure 3).

### 2.4. Comparison of Toxicity of AC-N, AC-T, and AC-T with Phosphate Limitation

**Figure 4:**
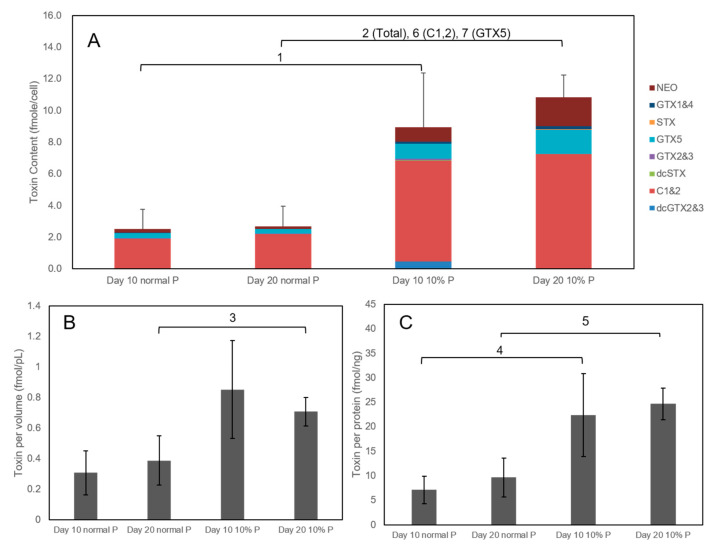
(**A**): toxin content and composition per cell of AC-T, (**B**): toxin content per volume of a cell of AC-T, and (**C**): toxin content per weight of the cellular protein of AC-T under normal and 10% phosphate conditions (10% P) after 10-day and 20-day post-inoculations. Significant differences between toxin contents of AC-T under normal P and 10% P in terms of moles toxin per cell (marked as 1 and 2), per cell volume (marked as 3), per cellular protein (marked as 4 and 5), as well as moles of C1 and 2 and GTX5 per cell (marked as 6 and 7, respectively) were verified using Student’s T-test with threshold *p* < 0.05.

Toxin contents of day 10 and day 20 cultures of AC-N under normal culturing conditions were analyzed and no toxin was detected. On the other hand, toxin contents of day 10 and day 20 cultures of AC-T with normal and 10% phosphate-limiting condition (i.e., with 10% normal phosphate provision) were shown in Figure 4. Toxin contents of AC-T cells on day 10 and day 20 cultures with normal provision of phosphate were 2.5 fmol/cell and 3.2 fmol/cell of PST respectively. However, with only 10% provision of phosphate, toxin contents of AC-T cells on day 10 and day 20 increased to 9.0 fmol/cell and 10.9 fmol/cell respectively (Figure 4A). Toxin contents of AC-T-10%P cells on day 10 (Figure 4) was already about 360% higher (^1^: *p* = 0.036) than that of normal AC-T cells, whereas that on day 20 was about 340% (^2^: *p* = 0.002) higher. Interestingly, cell size (Figure 3), as well as toxin contents of AC-T-10%P, was much higher than that of normal AC-T. As shown in Figure 4B, when toxin content was normalized by cellular volume, toxin contents in phosphate-limiting cells (0.71 fmol/pL) was still 180% higher (^3^: *p* = 0.04) in toxin contents compared to that of normal AC-T (0.39 fmol/pL) on day 20. This observation is more significant when toxin contents were normalized by total cellular proteins (Figure 4C), where toxin per proteins in AC-T-10%P (22.4 fmol/ng protein) was 310% higher than normal AC-T (7.1 fmol/ng protein) on day 10 (^4^: *p* = 0.041). The difference remained on day 20, where the toxicity of AC-T-10%P (24.7 fmol/ng protein) was 260% higher than normal AC-T (9.6 fmol/ng protein) (^5^: *p* = 0.007).

Overall toxin compositions of AC-T under two different phosphate cultivation conditions did not show obvious differences (Figure 4A), with C1 and C2 being the major STX derivatives, followed by neo-STX and GTX-5 as the second and third most abundant saxitoxin derivatives. Contents of C1 and 2 and GTX5 per cell in AC-T-10%P on day 20 (Figure 4A) were 400% (^6^: *p* = 0.004) and 520% (^7^: *p* = 0.018) higher than normal AC-T respectively on day 20, whereas contents of neo-STX and other derivatives did not show a significant difference in comparison of AC-T-10%P and normal AC-T on both day 10 and day 20. The ratio of the three most abundant toxins C1 and 2, neo-STX, and GTX5 were approximately 16:1:3 in normal AC-T, and 13:3:3 in AC-T-10%P on day 20, respectively. The composition of the other toxin derivatives detected was less than 2.5% of the total toxin contents.

### 2.5. Proteomes Comparison with 2-Dimensional Gel Electrophoresis (2-DE) Profiles and Protein Identification

**Figure 5:**
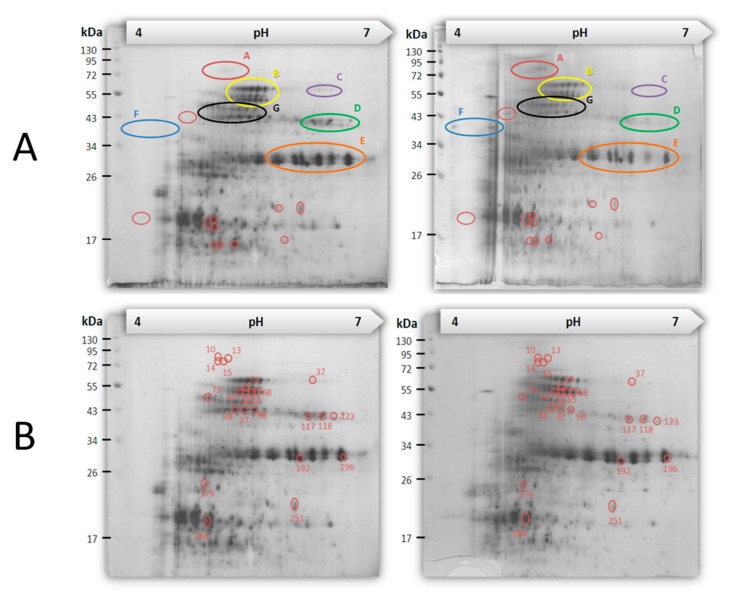
Representative gel pairs of 2-DE profiles of (**A**): AC-T (left) and AC-N (right), and (**B**): AC-T inoculated in normal phosphate condition (left) and that in phosphate-limited condition (right) on day 20. Circled gel regions are stained proteins found to be differentially expressed between two conditions.

We have two pairs of *A. catenella* cells with different amounts of cellular PSTs for comparison: (1) AC-T and AT-N, (2) AC-T and AC-T-10%P. With 2-DE, we intended to relate any differentially expressed proteins to their abilities to produce PST, AC-T, and AC-N cells in normal phosphate provision on day 20 of their growth were harvested and analyzed using 2-DE. AC-T cells in 10% phosphate provision were also harvested for 2-DE analysis. All samples were analyzed in triplicates. Over 300 matched protein spots were found among the 2-DE gels (Figure 5).

Between gels of AC-T and AC-N samples, 65 spots were found to be differentially expressed with at least 2-fold of difference (Figure 5A). Forty-four of the 65 differentially expressed spots in the AC-T/AC-N comparison were found to be more abundant in the AC-T cells and the remaining 21 were found to be less abundant in AC-T cells. When comparing 2-DE profiles of AC-T cells with normal phosphate and 10% phosphate provision, 22 differentially expressed spots with at least 2-fold changes were found (Figure 5B). All the 22 protein spots found in AC-T-10%P sample (i.e., with higher PST contents) were less abundant in comparison to AC-T cells in normal medium.

Identification of differentially expressed proteins was performed with a combination of MALDI-TOF-MS and LC-ESI-ion-trap mass spectrometry. In the comparison set between AC-T/AC-N, 23 protein spots representing 12 proteins were successfully identified (Table 1). Proteins that were more abundant in AC-T included ribulose 1,5-bisphosphate carboxylase/oxygenase II (RuBisCo II), methionine adenosyltransferase (MAT), chloroplast ATP synthase, nitrogen associated protein 50 (NAP50), nucleoredoxin, phosphoglycerate kinase (PGK), glyceraldehydes-3-phosphate dehydrogenase (G3PD), chloroplast ferredoxin-NADP(+) reductase, peridinin chlorophyll-a binding protein (PCP) apoprotein precursor, response regulator, light-harvesting protein and heat shock protein 70 (HSP70). None of the spots with decreased expression in AC-T compared to AC-N were successfully identified. On the other hand, in the AC-T and AC-T-10%P comparison, 18 of the 22 differentially expressed protein spots were identified and represented 10 proteins (Table 2). Interestingly, nine of these 10 proteins were also identified as more abundant in the AC-T cells in the AC-T/AC-N comparison. These proteins are chloroplast ATP synthase, NAP50, nucleoredoxin, PGK, G3PD, chloroplast ferredoxin-NADP(+) reductase, PCP apoprotein precursor, light-harvesting protein, and HSP70. Further, all the 10 proteins differentially expressed in the AC-T-10%P/AC-T comparison were less abundant in the AC-T-10%P cells. Table 3 summarizes all the proteins identified and their corresponding set of comparisons in which they were differentially expressed.

**Figure 6:**
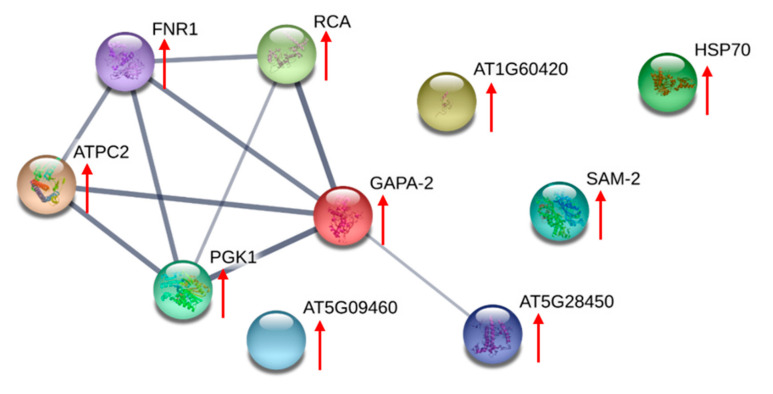
STRING protein-protein interaction network of differentially expressed proteins between AC-T and AC-N, using *Arabidopsis thaliana* as the model organism. The network joining the photosynthetic proteins including chloroplast ferredoxin-NADP(+) reductase (FNR1), peridinin chlorophyll-a binding protein apoprotein precursor (AT5G28450), glyceraldehyde-3-phosphate dehydrogenase (GAPA-2), ribulose 1,5-bisphosphate carboxylase/oxygenase II (RCA), phosphoglycerate kinase (PGK1), and chloroplast ATP synthase (ATPC2). All of them were found to be more abundant in AC-T cells (red arrows). The thicknesses of the lines joining the proteins refer to the confidence that they are interrelated.

**Figure 7:**
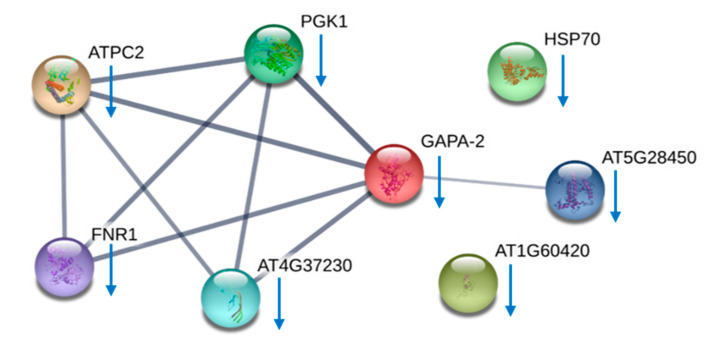
STRING protein-protein interaction network of differentially expressed proteins in AC-T under normal medium and phosphate-limited medium. A network form between chloroplast ferredoxin-NADP(+) reductase (FNR1), peridinin chlorophyll-a binding protein apoprotein precursor (AT5G28450), glyceraldehyde-3-phosphate dehydrogenase isoform 2 (GAPA-2), phosphoglycerate kinase (PGK1), oxygen-evolving enhancer-1 protein (AT4G37230), and chloroplast ATP synthase (ATPC2). All proteins in this network were down-regulated in AC-T-10%P cells (blue arrows).

Protein identities found in the two comparisons were further clustered using the string protein-protein interaction prediction algorithm. The network formed by these differentially expressed proteins found in AC-T/AC-N comparison and AC-T-10%P/AC-T is shown in Figure 6 and Figure 7 respectively. In Figure 6, proteins that were more abundant in AC-T cells compared to AC-N cells were mostly integrated into the same cluster as they are photosynthetic and energy production related proteins, meaning that these two inter-related cascades in AC-T cells were up-regulated compared to those in AC-N cells. However, the same network in Figure 7 had shown down-regulation in more toxic AC-T-10%P cells compared to less toxic normal AC-T cells.

Common protein identities found in both AC-T/AC-N and AC-T-10%P/AC-T comparisons were summarized in Table 3. Closer examination of the differentially expressed proteins revealed that most of them were related to photosynthesis and energy production. When compared to the non-toxic/less-toxic counterparts, why the toxic AC-T cells exhibited more of these enzymes expressed and why the more toxic AC-T-10%P exhibited less expression of the same sets of enzymes are certainly unknown. Nonetheless, it is important to note that photosynthesis and the ability for energy production ability are closely related to PST production.

To this end, we confirmed that phosphate-limiting conditions did induce more toxin production and that there were proteins differentially expressed after 20 days of phosphate limitation. It was found that between the two proteomic comparisons, expression of photosynthesis and energy-producing enzymes that are more abundant in toxic AC-T were substantially down-regulated in AC-T-10%P cells. Therefore, light availability and hence photosynthesis were involved in toxin production and/or regulation of AC-T cells. We then performed a continuous light and subsequent continuous dark exposure (photoperiod) experiment using the AC-T cells to study the effects of these modalities on PST production. However, it should be stressed that AC-T-10%P cells stopped to grow on day 7, and started to die out steadily from day 7 to day 20 in normal 12:12 light:dark cycle (Figure 2B). As a result, AC-T-10%P cells were not able to survive long enough and in substantial cell numbers in the photoperiod experiment with the additional changes of light and dark period as further environmental stress. Further, phosphate limitation would introduce additional variations on growth (in terms of cell number, cell volume, and cellular protein content) and probably various metabolic processes of AC-T cells in addition to those resulting from the change of light availability. Therefore, we only cultivated AC-T cells under normal phosphate provision in the subsequent photoperiod experiment.

### 2.6. Photoperiod Experiment to Investigate Effects on Toxicity of AC-T

**Figure 8:**
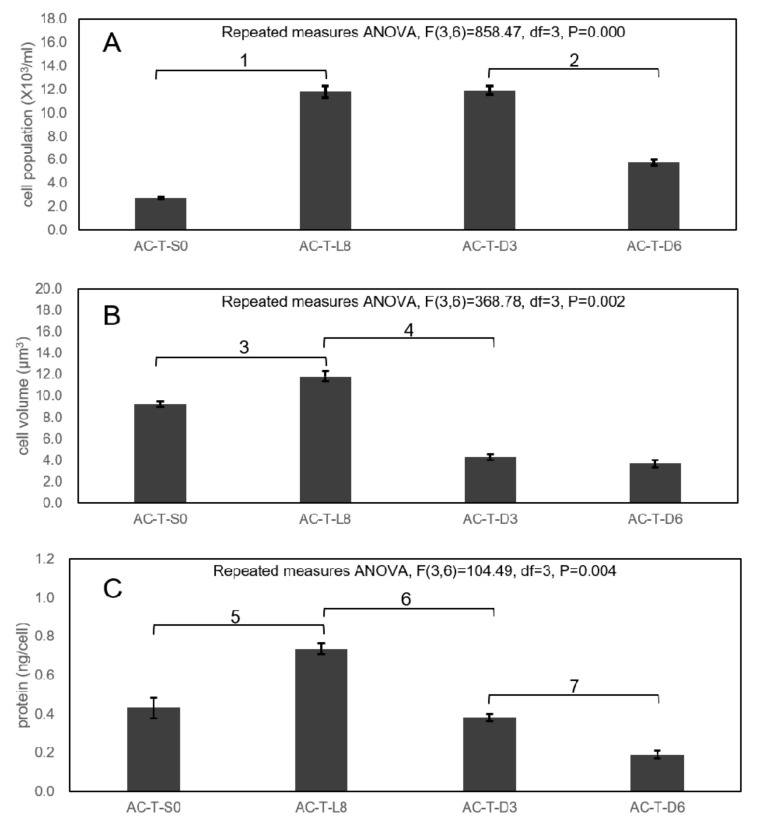
Variation in cell density per ml of culture (**A**), cell volume (**B**), and protein content per cell (**C**) of AC-T during the photoperiod experiment. Significant differences between AC-T cells during different photoperiods of the experiment in terms of cell populations (marked as 1 and 2), cell volumes (marked as 3 and 4), and cellular protein contents (marked as 5–7) were verified by repeated measures ANOVA and LSD post-hoc test with threshold *p* < 0.05.

**Figure 9:**
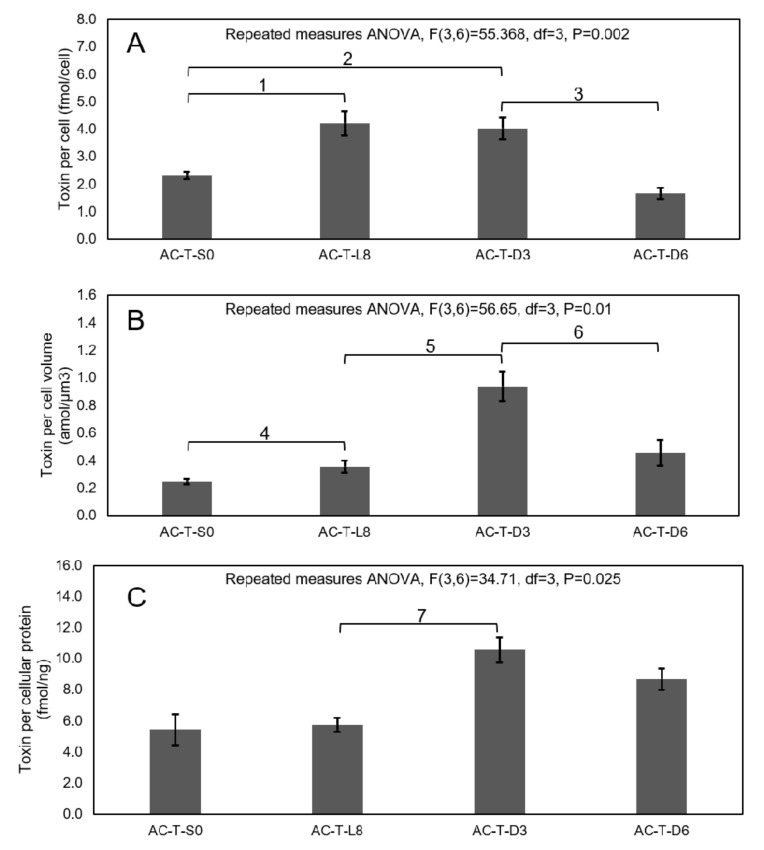
Variation in the mole of toxin per cell (**A**), per cell volume (**B**), and per cellular protein (**C**) of AC-T during the photoperiod experiment. Significant differences between toxin contents of AC-T cells during different photoperiods of the experiment in terms of moles of toxin per cell (marked as 1–3), per cell volume (marked as 4–6), and per cellular protein (marked as 7) were verified by repeated measures ANOVA and LSD post-hoc test with threshold *p* < 0.05.

After synchronizing AC-T cells in total darkness for 24 h (called AC-T-S0 sample), these cells were subjected to continuous light for 8 days (i.e., the AC-T-L8 sample), before subsequent exposure to total darkness for 3 days (AC-T-D3), 6 days (AC-T-D6), and 9 days (AC-T-D9) respectively. Changes of cell density, cell volume, and cellular protein content during the photoperiod experiment are shown in Figure 8. From the repeated measures ANOVA analysis, cell population density did not change on the AC-T-D3 sample after a significant increase from AC-T-S0 to AC-T-L8 (Figure 8A, ^1^: F(3,6) = 858.47, df = 3, *p* = 0.001). Cell volume (Figure 8B, ^3^: F(3,6) = 368.78, df = 3, *p* = 0.002) and cellular protein (Figure 8C, ^5^: F(3,6)=104.494, df=3, *p* = 0.024) also increased significantly from AC-T-S0 to AC-T-L8. However, cell volume and cellular protein content decreased significantly from (11.8 ± 0.4) × 10^3^ µm^3^ to (4.3 ± 0.3) × 10^3^ µm^3^(Figure 8B, ^4^: F(3,6) = 368.78, df = 3, *p* = 0.002), and from 0.74 ± 0.03 ng/cell to 0.38 ± 0.02 ng/cell (Figure 8C, ^6^: F(3,6) = 104.49, df = 3, *p* = 0.002, Figure 8C) respectively, after 3-day total darkness incubation. Toxicity of AC-T per cell in both AC-T-L8 and AC-T-D3 were significantly higher (Figure 9A, ^1^: F(3,6) = 55.368, df = 3; *p* = 0.008 and ^2^: *p* = 0.014 respectively) than that of the initial sample (AC-T-S0), whereas no significant difference was found between them. Toxin per cell volume of AC-T-L8 was also significantly higher than that of AC-T-S0 (Figure 9B, ^4^: F(3,6) = 56.65, df = 3, *p* = 0.018). Moreover, it should be stressed that AC-T-D3 and AC-T-D6 cells were much smaller in sizes than AC-T-S0 and AC-T-L8 samples (Figure 8B). Hence, when cell toxicity was normalized by cell volume (Figure 9B), cellular toxin concentration of AC-T-D3 was much higher than that of AC-T-L8 and AC-T-S0 (^5^: F(3,6) = 56.65, df = 3; *p* = 0.004 and *p* = 0.005 respectively). Similarly, cellular protein contents dropped by half after three days of darkness (Figure 8C), toxin contents expressed per weight of cellular proteins also increased significantly (Figure 9C, ^7^: F(3,6) = 34.71, df = 3, *p* = 0.01). When AC-T inoculums were further transferred to dark conditions for 9 days (AC-T-D9), there were no living cells found and none of the above parameters could be measured. Taken all together, AC-T cells in continuous light doubled in cell number, as well as the concomitant up-regulation of biosynthesis of proteins and toxins. When AC-T cells were shifted from exposure to continuous light for 8 days to continuous darkness for 3 days, the cells shrunk in size, and the major cytosolic content was reduced due to the lack of light and hence photosynthesis and associated energy production. Concurrently, the cellular toxin level remained constant from exposure of continuous light for 8 days (L8) transition to continuous darkness for 3 days (D3). From these results, it is thought that light, photosynthesis, and associated energy production drive cellular growth and PST production. Subsequently, when light is absent, vegetative growth and PST production stopped. In the initial 3 days of darkness, AC-T still survived despite its size reduction with concomitant loss of cellular protein. Hence, the total cellular toxin remained the same as that of AC-T-L8 (Figure 9A). When AC-T cells continued to be incubated in the dark from D3 to D6, cell population per ml of culture decreased by half (Figure 8A, ^2^: F(3,6) = 858.47, df = 3, *p* = 0.001), indicating the AC-T population was dying. Cellular volume and protein contents in D6 cells remained low (Figure 8B and C). Protein content of AC-T in D6 condition further decreased significantly when compared to D3 condition (Figure 8C, ^7^: F(3,6) = 104.494, df = 3, *p* = 0.011). Toxin per cell (Figure 9A) and toxin per cell volume (Figure 9B) decreased significantly from D3 to D6 condition (^3^: F(3,6) = 55.368, df = 3, *p* = 0.019, and ^6^: F(3,6) = 56.646, df = 3, *p* = 0.035, respectively), whereas toxin per cellular protein between AC-T-D3 and AC-T-D6 did not show significant difference. When the darkness was prolonged to 9 days, AC-T cells could not survive.

**Figure 10:**
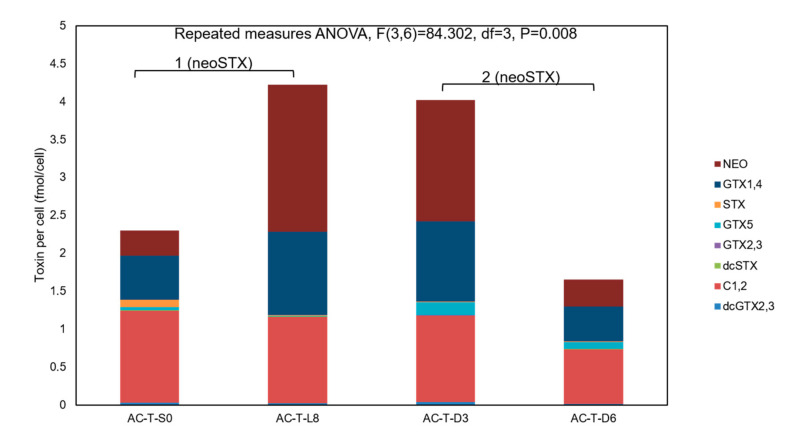
Variation in toxin composition per cell of AC-T during the photoperiod experiment. Significant differences between moles of neoSTX per cell (marked as 1 and 2) of AC-T during different photoperiods of the experiment were verified by repeated-measures ANOVA and LSD post-hoc test with threshold *p* < 0.05.

It is interesting to note that PST components of AC-T cells also varied throughout the photoperiod experiment. On day 0 and right after synchronization, C1 and C2 toxins were the dominant STX derivatives of AC-T cells (Figure 10), and the composition ratio of three most abundant toxins, neo-STX: GTX1 and 2: C1 and 2: others was approximately 3:5:10:1. These results are in line with AC-T cells grown in normal 12-12 light:dark cycles. However, after 8 days of continuous light (L8), proportions of neo-STX increased dramatically by six times (Figure 10, ^1^: F(3,6) = 84.302, df = 3, *p* = 0.005) and became the dominant derivatives (neo-STX: GTX1 and 2: C1 and 2: Others = 9:5:5:0.1). This change was not observed in the experiment of AC-T with 10% phosphate limitation, where cellular toxin content of AC-T-10%P also increased but overall toxin composition remained unchanged when compared to AC-T cells grown in normal culture medium (Figure 4A). The high proportions of neo-STX in AC-T cells remained after the cells were shifted from continuous light (L8) to total darkness for 3 days (D3), and the overall ratio of them was similar (neo-STX: GTX1 and 2: C1 and 2: Others = 8:5:5:1). This is also an indication that no new STX components were being synthesized in these 3 days of darkness. The proportion of neo-STX was found to be decreased in D6 conditions (Figure 10, ^2^: F(3,6) = 84.302, df = 3, *p* = 0.02). The results are in line with the observed decrease in cell density, cell volume, cellular protein, and toxin content.

## 3. Discussion

Besides apparently identical morphologies, our genetic verification work on the AC-T and AC-N showed a 97% homology in the ITS1-5.8s-ITS2 sequence regions. Growth curves as well as size measurements of the two strains are highly similar. By comparing their proteomes with 2-DE, a set of stringent identification criteria which includes a sequence of at least seven successive amino acids in each protein must be identified by either MALDI-TOF-MS or ESI-iontrap-MS to ensure high confidence on the identities of the proteins found to be expressed differentially. From Table 2 together with the results of STRING analysis (Figure 6), most of the proteins that were more abundant in the toxic strain compared to the non-toxic counterpart are photosynthetic-related proteins, including those in the light-harvesting complex and the Calvin cycle. In the same network of STRING interaction diagram, cellular energy-related enzymes including PGK, G3PD, and chloroplast ATP synthase were also more abundant in the toxic strain. All these results indicate that AC-T exhibited higher photosynthetic activity than the non-toxic AC-N. Our results are consistent with some reported previously [22,29]. However, it should be noted that there is one study which reported less abundance of light-harvesting complexes as well as photosystem I and II related proteins in a toxic strain of *A. catenella*, when compared to its non-toxic mutant [23].

Nitrogen-associated protein 50 (NAP50) was first found in the plastid of *Alexandrium affine* in our previous study [30]. NAP50 was down-regulated together with RuBisCo II when *A. affine* was cultivated in a nitrogen-depleted culture medium [30]. It was further suggested as a biomarker of nitrogen availability, as its transcript was also found to be down-regulated in *A. fundyense* grown in an ambient N-depleted environment [31]. It is also believed to be involved in metabolic pathways in plastids. As a result, higher expression of NAP50 in AC-T compare to AC-N also indicated potentially higher metabolic activity.

In addition to photosynthesis and energy-related proteins, methionine adenosyltransferase (MAT) was also found to be more abundant in AC-T than in AC-N. MAT involved in the synthesis of S-adenosylmethionine (SAM) with methionine and ATP in the methionine cycle in which methionine and homocysteine are interconverted. The product of MAT reaction, SAM, participates in a wide range of cellular processes. In PSTs producing dinoflagellates, SAM was reported to play a role in PST synthesis. The methyl group of methionine (through SAM) was a suspected precursor of the proposed STX synthetic pathway [32,33]. In the proposed mechanism, a side chain methyl group was added to the heterocyclic skeleton of the molecule by SAM after cyclization reactions. It should be noted that the unique starting gene, *sxtA* in cyanobacteria, was predicted as a SAM-dependent methyltransferase [16]. Another study on PSTs production reported that the gene of another SAM relating enzyme, adenosylhomocysteinase (AHS), was differentially expressed during toxin synthesis [17]. Hence, SAM may be regenerated at a higher rate and hence a higher expression of MAT in order to support toxin synthesis. However, although biosynthesis of SAM was reported to be potentially more active in toxic *A. catenella* compared to its non-toxic mutant [23], the key enzyme to drive the proposed pathway from SAM to STX skeleton, the predicted gene product of *sxtA* described above, was not identified in the same study. Moreover, seven other potential *sxt* gene product homologs identified by proteomic approaches in that study did not show any expressional difference between the toxic and non-toxic mutant [23]. In another study documenting proteomic changes of *A. catenella* at different stages of PST biosynthesis where *sxtA* homolog was identified for the first time, expression of *sxtA* also remained stable throughout the cell cycle regardless of fluctuation of cellular toxin contents throughout the cell cycle [34]. Owing to the wide range of biochemical roles of SAM as well as insufficient information acquired, the relationship between MAT and toxin synthesis needs to be investigated thoroughly.

Previous ecophysiological and ecological stoichiometric prediction studies reported that cellular toxin content of PST-producing *Alexandrium spp.* would be increased under phosphate limitation [7,8,9,13,17]. Similarly, from our study, growth of the cell density of AC-T stopped on day 7 (Figure 2) and the toxin content per cell increased by nearly 3.5 folds on day 20 with 10% of normal phosphate provision (Figure 4A). The most common explanation of this phenomenon was the accumulation of toxins produced inside undivided algal cells when there was insufficient phosphate for DNA synthesis [35] prerequisite to cell division. This was supported by the observation that cell volumes of AC-T cells increased under phosphate limitation. It was noteworthy to investigate whether the increase of cellular toxin content was solely because of a larger pool of accumulation or a result of a higher toxin production rate indeed. Therefore, in addition to cellular toxin content, toxin concentration per cell volume and toxin to cellular protein weight were studied. Notwithstanding, toxin content per cell volume and toxin per cellular protein of AC-T cells in phosphate-limited conditions were significantly higher than that of normal phosphate conditions on day 20 (Figure 4B and 4C). A higher toxin concentration inside algal cells indicated that the higher toxin content was not solely due to a larger pool (cell volume) from toxin accumulation, but a discrepancy between the rate of toxin synthesis and rate of cell biomass increase. These results reinforce the importance of investigating the protein expressional changes of AC-T cells under phosphate limitation and so the results of identified differentially expressed proteins in Table 2. There were 10 proteins identified which were differentially expressed between normal AC-T and that under phosphate-limited condition (Table 2). Most of the identified proteins in this comparison set (AC-T-10%P/AC-T) were those mentioned in the prior section discussing the AC-T/AC-N comparison (Table 1). The STRING diagram of this set of proteins formed a similar network to that of the previous one (Figure 7). PCP, PGK, G3PD, chloroplast ATP synthase, and ferredoxin-NADP(+) reductase are involved. Surprisingly, all these proteins play important roles in photosynthesis and were reported to decrease in abundance under phosphate limitation, although AC-T cells were found to be more toxic in such circumstances (Table 3). This is also in line with another study reporting that the same set of proteins involved in the Calvin cycle were also down-regulated in *A. catenella* cultures under phosphate depletion [36]. The only identified proteins which were not included in the previous comparison set were oxygen-evolving enhancer-1 (OEE) protein, which is also involved in the photosynthetic machinery. Surprisingly, although the Calvin cycle and ATP synthesis were reported to be down-regulated in the above study, expressions of OEE increased in *A. catenella* under phosphate limitation [36], which was contradictory to our result. OEE is located on the thylakoid lumen and has a stabilizing function for the tetra-manganese cluster for water oxidation in photosystem II [37]. It also serves as a protection of the reaction center of D1 protein from oxidizing actions of oxygen radicals. OEE is highly conserved in phototrophic organisms. A lower expression of OEE 1-2 precursor was found under phosphate limitation in the present study, indicating lower photosynthetic activities under the condition of phosphate stress.

From the proteomic comparison results, although conflicting trends of differential expression were seen in the more toxic strain/condition, it hinted that PST production may be related to photosynthesis and energy production mechanisms in the toxic AC-T cells. Hence, to investigate the relationship of photosynthesis with toxin synthesis, a photoperiod experiment was performed. That study showed that when there was light and hence photosynthesis, vegetative growth of the cells and production of PST took place. After the AC-T cells were shifted from 8-days of continuous light to 3-days of continuous dark, the absolute amount of cellular toxins was maintained. This cellular amount of PST started to decline after 6 days of darkness (Figure 8). Similar results were reported in *Alexandrium minutum*, that STX content disappeared completely after incubating the culture in the dark for 22 days [35]. This change of toxicity after continuous darkness further indicated that light energy is crucial for toxin biosynthesis. However, the most important lesson from this photoperiod experiment is that after continuous 8 days of light and then 3 days of total darkness: (1) while the cellular toxin content remained constant after 3 days, (2) the cells shrunk and the cellular protein contents decreased. Furthermore, when there was no light (total darkness for 3 days), no new toxin was synthesized. Shrinking of the cells and loss of cellular proteins gave the apparent increase in cellular toxicity, both in terms of toxin per cell volume and toxin per cellular protein weight. With the deprivation of light for three more days, on day 6 of total darkness, total cellular toxin content, toxin concentration, and total cellular proteins decreased. Most AC-T cells died after 9 days of total darkness. Hence, the decrease in total cellular toxins after 6 days of total darkness was probably due to leakage and loss of cellular integrity as there was a substantial loss of cellular proteins and cellular shrinkage.

Also from our results, the growth of AC-T under phosphate limitation was retarded after several days of cultivation and photosynthetic activities decreased concomitantly (Figure 2B). In a published study, the fluorescence-based photochemical efficiency of *A. minutum* decreased under phosphate limitation [9]. It was suggested that such a drop was the result of the reduction of ATP acquisition by the cells, establishing a pH gradient across the thylakoid membranes and hence nonphotochemical quenching. Regarding toxicity, in the study by Lippemeier and coworkers [9] as well as the present study, cellular toxin contents increased in PO_4_-limited *Alexandrium*, showing an inverse relationship with photosynthetic activities. The relationship between photosynthesis and toxin content might suggest that light availability could affect the toxin synthesis of dinoflagellates. As light availability also affects photosynthetic activity, toxin production at continuous light may be a consequence of changing the photosynthetic rate in *A. catenella*. However, the inverse relationship of photosynthetic-related protein expression and cellular toxin content in phosphate-limited AC-T cells was contradictory with the suggestion that the product or process of photosynthetic capabilities is required for toxin synthesis, as suggested in the comparisons of AC-T versus AC-N. It also disagreed with the suggestion that the ability of toxin synthesis is solely metabolism-driven. This conflicting phenomenon suggests a hypothesis that there are different levels of control that regulates PST production. These regulatory mechanisms could be light-dependent or light-independent. Non-toxic AC-N is intrinsically unable to carry out toxin synthesis, but toxic AC-T seems to have the genetic machinery to produce PSTs supported by additional photosynthetic activity compared to AC-N. Furthermore, the results of photoperiod experiments stressed the relevance of light availability on AC-T to produce toxins, which further indicated the involvement of light and hence photosynthesis on toxin production of *A. catenella*. Certainly, further investigation is needed to reveal the relationship between toxin production/regulation light availability and phosphate supply. Taken all together, the present study has demonstrated that PST-production in *Alexandrium catenella* is likely regulated by multiple layers of control mechanisms, involving the changes of expressions of photosynthetic and energy-producing enzymes.

## 4. Materials and Methods

### 4.1. Chemicals and Materials

Except stated otherwise, all chemicals used were from Sigma-Aldrich (St. Louis, MO, USA) and are at least of analytical grade. Water used was of ultra-pure grade and purified using a Milli-Q Type-1 system (Millipore, Burlington, MA, USA). All solvents used were at least of HPLC grade. Nuclease-free reagents and apparatus were used in DNA-based identification procedures. Safe-lock micro-centrifuge tubes (Eppendorf, Hamburg Germany) and Diamond pipette tips (Gilson, Middleton, WI, USA) were used in all steps of the proteomic experiments. Except specified otherwise, experiments were designed and completed with biological triplication.

### 4.2. Dinoflagellates Cultures and Cultivating Conditions

Monoclones of toxic *Alexandrium catenella* (AC-T) was isolated from the Silver Mine Bay of Hong Kong in March 2009. The non-toxic *A. catenella* (AC-N) was cultivated from a single-cell isolate of an *Alexandrium* culture (CS-319) obtained from the Australian National Algae Culture Collection (ANACC, Canberra Australia). Non-toxic *A. tamarense* CCMP116 (denoted as AT01) was obtained from the National Center of Marine Algae and Microbiota (NCMA, East Boothbay, ME, USA). L1-Si seawater-based medium was used for all cultures [37]. All cultures were grown in a white fluorescent light chamber (Sanyo, Moriguchi, Japan). The temperature and light intensity of the chamber was kept at 22 °C and 7 μmole m^−2^ s^−1^ respectively, under a 12:12 h light:dark cycle. Experimental cultures were inoculated with vegetative cells from stock cultures during the mid-exponential growth phase (~10,000 cells per mL) after centrifugation at 360× *g* for 5 min and then started with 1000 cells per ml by concentration and 500 mL by volume on day 0. Phosphate limited AC-T cultures were inoculated in the same condition except only 3.62 μM of NaH_2_PO_4_ in the Li-Si medium (i.e., 10% of the original Li-Si medium, which contains 36.2 μM of NaH_2_PO_4_) of cells were harvested by centrifugation at 2700× *g* for 5 min. Cell pellets were stored in −80 °C for further investigation. Statistical analysis of cell number comparison of AC-T vs. AC-N, as well as AC-T under normal phosphate vs. phosphate limited conditions, were performed using SPSS 25 (IBM, Armonk, NY, USA). Comparisons made between two independent variables were analyzed using independent Student’s T-test with a significant threshold *p* < 0.05. The Shapiro-Wilk test was used to confirm the normality of the dataset prior to T-test calculation, whereas F-test was used to deduce the equality of variance of the datasets.

### 4.3. DNA-Based Identification

The ribosomal gene (partial LSU) and Internal Transcribed Spacer (ITS) sequences were used to verify the identities of the dinoflagellate cultures. Cells from 200 mL of the *Alexandrium* cells were harvested for DNA-based identification. DNA from the harvested biomass was extracted using the High Pure PCR Template Preparation Kit (Roche, Switzerland). The ITS 1 and 2 regions together with the 5.8S ribosomal DNA was amplified by polymerase chain reaction (PCR) with primers ITSA (5′ CCGGATCCAAGCTTTCGTAACAAGGHTCCGTAGGT 3′) and ITSB (5′ CCGGATCCGTCGACAKATGCTTAARTTCAGCRGG 3′) [38]. Partial 28S ribosomal sequence was amplified with primers 28S-F (5′ GGTGGAAAWTRAMCCAAMDGGG 3′) and 28S-R (5′ GTGTTTCAAGASGGGTCARRCA 3′). Amplification was performed by 35 replications of the following cycle: denaturing (94 °C; 40 s), annealing (50 °C; 40 s), and elongation (72 °C; 60 s). The whole amplification process was ended with 72 °C for a further 10 min. Purified PCR products were cloned into pGEM-T easy vectors (Promega, Madison, WI, USA). Cloned plasmids were extracted by QIAprep^®^ Spin Miniprep Kit (Qiagen, Hilden, Germany). DNA sequences were obtained using Sanger sequencing services provided by Beijing Genomic Institute (BGI, Shenzhen, China). Sequences obtained were aligned using ClustalX [39] and wrtr also searched through the BLAST algorithm [40] against the NCBI nucleotide database [41].

### 4.4. Cell Counts and Growth Curves

Algal densities in the cultures were determined using the Sedgewick-Rafter counting chamber after fixing with Lugol’s solution and then examined under a light microscope. Specific growth rates (µ) was calculated using the following formula where N_2_ and N_1_ are the cell density in the form of cell numbers per ml at their corresponding time point, t_2_ and t_1_, respectively:

$$µ=\frac{ln\;N_{2}-ln\;N_{1}}{t_{2}-t_{1}}$$

### 4.5. Cell Size Measurement

Cell volumes were estimated following a previously reported protocol [28]. In brief, the diameter (d) and height (h) of the cells were recorded with the Axia Vert A1 microscope system (Carl Zeiss, Oberkochen, Germany). Mean cell volumes in μm^3^ were calculated from 30 random measurements. The cell volumes (V) were calculated with the following formula:

$$V=\frac{\pi }{6}\times d^{2}\times h$$

Student’s *T*-test of cell volume comparisons of AC-T vs. AC-N as well as AC-T under normal phosphate vs. phosphate limited conditions were performed as described in Section 4.2.

### 4.6. Cellular Protein Analysis

One hundred ml of *Alexandrium* cells were harvested by centrifugation at 2700× *g* for 5 min. Pellets were resuspended in lysis buffer (7 M urea, 2 M thiourea, 4% CHAPS, 1% dithiothreitol). Cells in the suspension were lysed by ultrasonication using a UP 200s ultra-sonicator (Dr.Hielscher, Berlin, Germany) with 15 s/15 s pulse intervals in the total process of 3 min. Cell debris was removed by centrifugation, and the protein contents in the supernatant were measured by Bradford’s protein assay following the manufacturer’s protocol (BioRad, Hercules, CA, USA) using bovine serum albumin (BSA) as a calibration standard. Student’s T-test of cellular protein content comparisons of AC-T vs. AC-N as well as AC-T under normal phosphate vs. phosphate limited conditions were performed as described in Section 4.2.

### 4.7. Toxin Analysis

Toxin levels in the dinoflagellate samples were analyzed by a UPLC system (Waters, Milford, MA, USA) coupled with a pre-column oxidation method developed previously [42]. Endogenous toxins in samples were first extracted with 1mL 0.05 M acetic acid prior to ultrasonication as described in Section 4.6. The samples were then centrifuged at 15,000× *g* for 5 min. Supernatants were stored under −20 °C before use. Samples were divided into 2 portions and each portion was oxidized with one of two different oxidation methods. Samples intended to measure their contents of STX, dcSTX, GTX2+3, GTX5, and C1+2 were oxidized by 10% hydrogen peroxide solution in 1 M sodium hydroxide for 5 min before being stopped by 5% acetic acid. Samples intended to measure the contents of neo-STX and GTX1+4 were oxidized by periodate oxidant prepared with 0.1 M periodic acid, 0.1 M ammonium formate, and 0.1 M sodium phosphate dibasic before being stopped by 7% acetic acid. After the reactions, oxidized toxin solutions were filtered into sample vials with 0.2 μm PTFE syringe filters. Separation and quantification of oxidized PST derivatives were achieved by reversed-phase UPLC system with a 2.1 × 100 mm HSS T3 column (Waters, Milford, MA, USA). Two mobile phases were used for the UPLC analysis: 0.1 M ammonium formate (pH 6.0, buffer A) and 0.1 M ammonium formate with 5% acetonitrile (pH 6.0, buffer B). The flow rate was kept at 0.5 mL/minute and the column temperature was kept at 35 °C. The elution gradient program was divided into 3 steps with different ratios of buffer A to buffer B: 95:5 from the 0 to 2.5 min, 70:30 from the 2.5 to 5 min, and 100:0 from the 5 to 6 min. Separated toxins were detected by fluorometer with λ_ex_ 340 nm and λ_em_ 395 nm. Peak areas in the UPLC chromatograms were measured and the amounts of cellular toxins were quantified with the standard curves. Intracellular toxin contents were generally expressed as fmol/cell after summing up all the derivatives measured. Student’s T-test of toxin content comparisons of AC-T vs. AC-N as well as AC-T under normal phosphate vs. phosphate limited conditions were performed as described in Section 4.2.

### 4.8. Protein Extraction and 2-Dimensional Gel Electrophoresis (2-DE)

Protein samples were extracted from the harvested cells with the use of the TriPure Isolation kit (Roche, Basel, Switzerland). The protocol of the extraction procedure was optimized for sample preparation of dinoflagellates for 2-DE [43]. Extracted proteins were dissolved in lysis buffer (7 M urea, 2 M thiourea, 4% CHAPS, 1% dithiothreitol) at pH 8.5 and stored in −20 °C until further studies.

2-DE was firstly performed by rehydration containing either 80 μg protein for silver staining or 1 mg protein for Coomassie blue staining with 18 cm IPG strips pH 4-7 (BioRad, Hercules, CA, USA) for 16-20 h at room temperature using rehydration buffer (7 M urea, 2 M thiourea, 4% CHAPS, 5% glycerol, 10% isopropanol) added with small amount of dithiothreitol (DTT) and 1% *v/v* of IPG buffer pH 4-7. Isoelectric focusing was then followed using Protean-IEF cell (BioRad, Hercules, CA, USA) according to the following protocol: 500 V for 3 h, 1000 V for 6 h, 8000 V for 3 h, and finally with a total of 120,000 voltage hours. The gel strips were incubated for 15-min with the equilibration buffer (50 mM Tris pH 8.8, 6 M urea, 30% glycerol, 2% SDS, and a trace amount of bromophenol blue) containing 1% DTT and 2.3% iodoacetamide (IAA) respectively. The second dimension of SDS-PAGE was performed in 12% polyacrylamide gel using the PROTEAN^®^ II xl cell (BioRad, Hercules, CA, USA) under voltage of 35 mA/gel. Subsequently, the gels were fixed in 10% *v/v* acetic acid and 40% *v/v* methanol.

### 4.9. Gel Staining and Image Analysis

Visualization of proteins was either performed with silver staining or Coomassie blue staining. Silver staining was performed in 3 steps. Fixed gels were sensitized for 30 min (8 mM sodium thiosulphate and 30% *v/v* methanol). Followed by rinsing with deionized water, the gels were incubated in 14.7 mM silver nitrate solution for 20 min. After washing out silver nitrate with deionized water, a 0.24 M sodium carbonate solution in 0.004% *v/v* formaldehyde was used for developing images and the developing process was stopped by 35 mM EDTA solution when the stain was adequate.

Coomassie blue staining was performed by 1-h staining and several rounds of destaining, using the 0.1% Coomassie blue stain and destain solution (5% methanol and 7% acetic acid) respectively.

The stained 2-D gels were scanned before image analyses by the Melanie III software (GeneBio, Geneva, Switzerland). Each gel pairs were automatically aligned by Melanie, followed by a manual adjustment of alignments based on commonly visible spots, such as those in region B, E and G circled on Figure 5. Intensities of spots were then normalized using total volume normalization method by the software, and eventually generated a series of visualized bar charts showing the mean and mean square derivation (MSD) of the intensity of each aligned spots (please refer to Supplementary Materials: Figures S1 and S2). Spots with mean intensity differences > 2 folds and with two un-overlapped MSD bars were classified as differentially expressed proteins and were picked for protein identification.

### 4.10. In-Gel Digestion

Gel-plugs containing the proteins of interest pinpointed by 2-D gel image analysis were excised from the Coomassie blue-stained gel for in-gel tryptic digestion before analyzed by matrix-assisted laser desorption/ionization time-of-flight (MALDI-TOF-TOF) mass spectrometry. The excised Coomassie blue-stained gel plugs with about 1 mm × 1 mm size were washed with 25 mM ammonium bicarbonate (NH_4_HCO_3_) solution in 50% acetonitrile for three times with 10 min in each washing step. After dehydrating with 100% acetonitrile (ACN), the gel pieces were reduced and alkylated with 10 mM DTT (55 °C for 45 min) and 55 mM IAA (room temperature; in dark for 30 min) respectively. The gel plugs were dried again by 100% ACN after washed with 25 mM NH_4_HCO_3_ solution in 50% ACN. The gel pieces were then rehydrated with about 10 μL of NH_4_HCO_3_ solution containing 60 ng of sequencing grade trypsin (Promega, Madison, WI, USA) before being incubated in 37 °C overnight. Digested peptides inside the gels were eluted with 0.1% trifluoroacetic acid (TFA) in 50% ACN with the aid of water bath ultra-sonication. The peptide solution was then dried SpeedVac (Labconco, Kansas City, MO, USA). Dried peptide fragments were re-suspended in 2 μL of 0.1% TFA with ACN (2:1).

### 4.11. MALDI-TOF-MS Analysis and N-terminal Sulfonation

Each spot on the anchor-chip was firstly coated with 2 mg/mL α-cyano-4-hydroxycinnamic acid (HCCA) in 0.1% TFA with ACN (2:1) prior to adding samples. The resuspended peptide samples were then introduced onto a stainless steel anchor plate and were allowed to dry again. Followed with brief washing of 0.1% TFA, samples were subsequently recrystallized with 0.5 μL of a mixture of ethanol, acetone, and 0.1% TFA in a volume ratio of 6:3:1. Samples were then ready for MALDI-TOF MS analysis with Autoflex III (Bruker, Billerica, MA, USA). Reflector mode over a mass range of 700–3000 m/z was used after calibration to a mass tolerance of 150 ppm using peptide calibration standard II (Bruker, Billerica, MA, USA) as external calibrants. Peptide mass fingerprints (PMF) were generated from the combined spectra of 1000 shots of each sample. Peptide ions visualized in the spectra were further fragmented into its constituent amino acids with post-source decay in the LIFT mode at 19.0 kV. The MS/MS spectra generated were analyzed by the de novo sequencing function of Biotools 3.2 (Bruker, Billerica, MA, USA) to construct its amino acid sequences. The amino acid sequence tags with at least 7 successive amino acids of unknown proteins were searched against the NCBI non-redundant database [41] as well as our in-house transcriptome database built using AC-T cells (data not shown) using Mascot Server 2.2 (Matrix Science, London, UK). Precursor tolerance and MS/MS tolerance were set to be 150 ppm and 0.3 Da respectively. Parent charge of ions was set to be +1. Cysteine carbamidomethylation was set as fixed modification and methionine oxidation was set as variable modification. Positive identification was accepted when protein identified with MOWSE scores probabilities less than 0.05.

De-novo peptides sequencing was facilitated with the aid of N-terminal sulfonation in order to form y-ions predominately in the MS/MS spectra [44]. Sulfonation was performed by incubating the dehydrated tryptic peptides with 10 mg/mL 4-sulfophenyl isothiocyanate (SPITC) in 20 mM sodium bicarbonate at pH 9.5 in 55 °C for 30 min, after trypsin digestion. After stopping with 0.5% TFA, sulfonated peptide fragments mixture was extracted using C-18 zip-tips (Millipore, Burlington, MA, USA) following the manufacturer provided protocol. Eluted peptides were directly targeted onto the anchor plate in which the matrix had been already applied onto the corresponding spots. Successfully sulfonated peptides would have an increased mass of 215 Da. MALDI-TOF-TOF analysis was performed with the sulfonated peptides to generate the MS/MS spectra. Followed-up de novo sequences were then analyzed with Biotools 3.2 (Bruker, Billerica, MA, USA).

### 4.12. LC-ESI-iontrap Analysis

The tryptic peptide mixture of protein of interest (which was suspended in 5 μL of 2% ACN and 0.1% formic acid) was loaded into a 15-cm nanoflow C18 column (Dionex, Sunnyvale, CA, USA). The column was equilibrated earlier with 0.1% formic acid. Peptides eluted with elution buffer (20% H2O, 80% acetonitrile and 0.001% formic acid) at a flow rate of 450 nL/min and column temperature 40 °C were directly introduced into an HCT-Ultra ESI-iontrap mass spectrometer (Bruker, Billerica, MA, USA). The peptide ions were acquired by positive mode with 1400 V of capillary voltage and 80–120 nA current in the iontrap. The temperature was set at 150 °C and the accumulation time for the peptide ions in the iontrap was set to be 50 ms. Nitrogen was introduced at a flow rate of 6 L/min. MS/MS spectra obtained were analyzed with Data Analysis 3.4 (Bruker, Billerica, MA, USA) and Biotools 3.2 to deduce the amino acid sequences. Finally, putative identification of proteins of interest was performed by searching against the NCBInr and in-house AC-T transcriptome database with similar criteria as MALDI-TOF-TOF analysis except the charged states were set to 2+, 3+, and 4+, as well as 1.2 Da of precursor tolerance and 0.6 Da of MS/MS tolerance.

### 4.13. STRING Analyses

Interaction between identified proteins was investigated by STRING v11.0, an online database of known and predicted protein-protein interactions [45]. Two sets of identified differentially expressed proteins were loaded into the program separately and the confidence level was set to medium level (score > 0.4). The calculation using text-mining is unchecked such that only interactions with experimental supports were investigated. As there are no dinoflagellate databases available in the String algorithm, *Arabidopsis thaliana* was set to be the model organism.

### 4.14. Photoperiod Experiments

A flask of 3L AC-T culture after 7-days post-inoculation was sub-cultivated into 3 replicates in 3L cultures with 3000 cell/mL and the cells were synchronized in darkness in 25 °C for 24 h. The cultures then faced 17-days of photoperiod experiments with 8-days of 24-h light, followed by 9-days of 24-h dark in 25 °C. Cell samples were taken right after synchronization (AC-T-S0), after 8-days of 24-h light (day 8 post-synchronization, AC-T-L8), after 3-days of 24-h darkness (day 11 post-synchronization, AC-T-D3), after 6-days of darkness (day 14 post-synchronization, AC-T-D6) and after 9-days of 24-h darkness (day 17 post-synchronization, AC-T-D9) were harvested for cell numbers, cell volume, cellular protein content and toxin content measurement. Data acquired from this experiment were analyzed using repeated-measures ANOVA test with Greenhouse-Geisser correction [46,47]. Comparisons with ANOVA *p*-value < 0.05 (i.e., a significant difference existed between time points) were subjected to post-hoc comparison using the least significant difference (LSD) test with a significant threshold = 0.05.

## Figures and Tables

**Table 1:** Differentially expressed proteins identified between AC-T and AC-N.

Significantly Matched Proteins	Differential Expression Ratio (AC-T: AC-N)	NCBI Accession no.	Spot Number	pI Value (pI)	Molecular Weight (kDa)	Mascot Score	Identified by
*(More abundant in AC-T)*							
ribulose 1,5-bisphosphate carboxylase/oxygenase II	5.2:1	AAO13045.1 AAO13070.1	27	5.4	60	Blastp E-value: 1.00E-04 (de novo sequencing)	MALDI-TOF
	5.2:1		28	5.4	60		
	4.7:1		31	5.5	60		
	4.1:1		32	5.6	60		
	3.2:1		33	5.7	60		
methionine adenosyltransferase	5.9:1	NP_001035469.1	38	6.6	60	141	MALDI-TOF
chloroplast ATP synthase gamma submit	2.5:1	AAW79296.1	54	5.4	55	197	ESI-iontrap
nitrogen associated protein 50	2.2:1	ACV60537.1	63	5.4	50	232	ESI-iontrap
	2.2:1		65	5.5	50		
nucleoredoxin	Only detected in AC-T	KOO32230.1	73	5.0	48	167	ESI-iontrap
phosphoglycerate kinase	5.5:1	OLP97413.1	96	5.3	43	332	ESI-iontrap
	4.5:1		97	5.4	43		
	2.9:1		98	5.5	43		
glyceraldehyde-3 phosphate dehydrogenase	7.9:1	ABI14256.1	104	6.5	42	110	MALDI-TOF
chloroplast ferredoxin-NADP(+) reductase	4.7:1	AAW79314.1	117	6.4	42	461	ESI-iontrap
	7.0:1		118	6.6	42		
	8.2:1		123	6.7	42		
peridinin chlorophyll-a binding protein apoprotein precursor	3.0:1	AFH88375.1	175	6.5	30	1343	ESI-iontrap
response regulator	2.4:1	*WP_011605320.1*	257	5.0	19	67	MALDI-TOF
light harvesting protein	2.0:1	CBI83417.1	266	5.0	19	650	ESI-iontrap
heat shock protein 70	6.1:1	AAM02973.2	10	5.2	80	487	ESI-iontrap
	7.7:1		15	5.3	78		
	6.5:1		14	5.2	78		

**Table 2:** Differentially expressed proteins identified between AC-T under normal phosphate and 10% phosphate limitation.

Significantly Matched Proteins	Differential Expression Ratio (AC-T:AC-T-10%P)	NCBI Accession No.	Spot Number	pI Value (pI)	Molecular Weight (kDa)	Mascot Score	Identified by
*(More abundant in AC-T)*							
heat shock protein 70	1:3.7	AAM02973.2	10	5.2	80	487	ESI-iontrap
	1:7.2		15	5.3	78		
	1:2.5		14	5.2	78		
chloroplast ATP synthase	1:3.7	AAW79296.1	54	5.4	55	197	ESI-iontrap
nitrogen associated protein 50	1:5.9	ACV605371.	63	5.4	50	232	ESI-iontrap
	1:2.1		65	5.5	50		
	1:2.0		66	5.5	50		
nucleoredoxin	1:3.7	KOO32230.1	73	5.0	48	167	ESI-iontrap
phosphoglycerate kinase	1:7.3	OLP97413.1	96	5.3	43	332	ESI-iontrap
	1:7.7		97	5.4	43		
	1:6.1		98	5.5	43		
10lyceraldehyde-3 phosphate dehydrogenase	1:2.9	ABI14256.1	104	6.5	43	110	MALDI-TOF
chloroplast ferredoxin-NADP(+) reductase	1:3.0	AAW79314.1	117	6.4	42	461	ESI-iontrap
	1:2.9		118	6.6	42		
	1:2.4		123	6.7	42		
peridinin chlorophyll-a binding protein	1:4.2	AFH88375.1	192	6.3	28	407	ESI-iontrap
oxygen-evolving enhancer-1 protein	1:3.5	OLQ01973.1	196	6.8	29	334	ESI-iontrap
light harvesting protein	1:2.2	AAM02973.2	266	5.0	19	650	ESI-iontrap

**Table 3:** Summary of the differentially expressed proteins commonly found in two sets of comparisons. In the table, photosynthesis-related proteins and cellular energy-related proteins were up-regulated in the toxic AC-T cells (AC-T ↑) in the AC-T vs. AC-N comparison, whereas in the AC-T-10%P vs. AC-T comparison, they were down-regulated in the more toxic samples (AC-T-10%P ↓).

Identified Proteins	General Functions	Differentially Expressed between AC-T and AC-N	Differentially Expressed between AC-T and AC-T-10%P
Ribulose 1,5-bisphosphate carboxylase/oxygenase II	Photosynthesis related	AC-T ↑	Not differentially expressed
Chloroplast ATP synthase			AC-T-10%P ↓
Chloroplast ferredoxin-NADP(+) reductase			
Light-harvesting protein			
Peridinin chlorophyll-a binding protein apoprotein precursor			
Oxygen-evolving enhancer-1 protein		Not differentially expressed	
Glyceraldehydes-3-phosphate dehydrogenase	Cellular energy-related	AC-T ↑	AC-T-10%P ↓
Phosphoglycerate kinase			
Nitrogen associated protein 50	Unknown		
Nucleoredoxin	Signaling related		
Heat shock protein 70	Environmental stress-related		

## Supplementary Materials

The following are available online at https://www.mdpi.com/2072-6651/12/8/477/s1, Figure S1: mean and mean square derivation (MSD) of some aligned spots found in AC-T/AC-N comparison, Figure S2: mean and MSD of some aligned spots found in AC-T-10%P/AC-T comparison.

## References

[1] Garcıa C., del Carmen Bravo M., Lagos M., Lagos N. (2004). Paralytic shellfish poisoning: Post-mortem analysis of tissue and body fluid samples from human victims in the Patagonia fjords. Toxicon.

[2] Sobel J., Painter J. (2005). Illnesses caused by marine toxins. Clin. Infect. Dis..

[3] Deeds J.R., Landsberg J.H., Etheridge S.M., Pitcher G.C., Longan S.W. (2008). Non-traditional vectors for paralytic shellfish poisoning. Mar. Drugs.

[4] Lim P.-T., Ogata T. (2005). Salinity effect on growth and toxin production of four tropical Alexandrium species (Dinophyceae). Toxicon.

[5] Ichimi K., Suzuki T., Ito A. (2002). Variety of PSP toxin profiles in various culture strains of Alexandrium tamarense and change of toxin profile in natural A. tamarense population. J. Exp. Mar. Biol. Ecol..

[6] Caruana A.M., Amzil Z. (2018). Microalgae and Toxins. Microalgae in Health and Disease Prevention.

[7] Boyer G., Sullivan J., Andersen R., Harrison P., Taylor F. (1987). Effects of nutrient limitation on toxin production and composition in the marine dinoflagellate Protogonyaulax tamarensis. Mar. Biol..

[8] Anderson D., Kulis D., Sullivan J., Hall S. (1990). Toxin composition variations in one isolate of the dinoflagellate *Alexandrium fundyense*. Toxicon.

[9] Lippemeier S., Frampton D.M., Blackburn S.I., Geier S.C., Negri A.P. (2003). Influence of phosphorus limitation on toxicity and photosynthesis of Alexandrium minutum (Dinophyceae) moniteres by in-line detection of variable chlorophyll flourescence 1. J. Phycol..

[10] Leong S.C.Y., Murata A., Nagashima Y., Taguchi S. (2004). Variability in toxicity of the dinoflagellate *Alexandrium tamarense* in response to different nitrogen sources and concentrations. Toxicon.

[11] Lim P.-T., Leaw C.-P., Kobiyama A., Ogata T. (2010). Growth and toxin production of tropical Alexandrium minutum Halim (Dinophyceae) under various nitrogen to phosphorus ratios. J. Appl. Phycol..

[12] Xu J., Ho A.Y., He L., Yin K., Hung C., Choi N., Lam P.K., Wu R.S., Anderson D.M., Harrison P.J. (2012). Effects of inorganic and organic nitrogen and phosphorus on the growth and toxicity of two Alexandrium species from Hong Kong. Harmful Algae.

[13] Van de Waal D.B., Smith V.H., Declerck S.A., Stam E.C., Elser J.J. (2014). Stoichiometric regulation of phytoplankton toxins. Ecol. Lett..

[14] Kellmann R., Michali T.K., Neilan B.A. (2008). Identification of a saxitoxin biosynthesis gene with a history of frequent horizontal gene transfers. J. Mol. Evol..

[15] Moustafa A., Loram J.E., Hackett J.D., Anderson D.M., Plumley F.G., Bhattacharya D. (2009). Origin of saxitoxin biosynthetic genes in cyanobacteria. PLoS ONE.

[16] Stüken A., Orr R.J., Kellmann R., Murray S.A., Neilan B.A., Jakobsen K.S. (2011). Discovery of nuclear-encoded genes for the neurotoxin saxitoxin in dinoflagellates. PLoS ONE.

[17] Taroncher-Oldenburg G., Anderson D.M. (2000). Identification and characterization of three differentially expressed genes, encoding S-adenosylhomocysteine hydrolase, methionine aminopeptidase, and a histone-like protein, in the toxic dinoflagellate *Alexandrium fundyense*. Appl. Environ. Microbiol..

[18] Cho Y., Ogawa M., Hirota M., Oshima Y. (2011). Effects of mitomycin C and colchicine on toxin production and cell cycle regulation in the dinoflagellate *Alexandrium tamarense*. Harmful Algae.

[19] Zhang S.-F., Zhang Y., Lin L., Wang D.-Z. (2018). iTRAQ-based quantitative proteomic analysis of a toxigenic dinoflagellate Alexandrium catenella and its non-toxigenic mutant exposed to a cell cycle inhibitor colchicine. Front. Microbiol..

[20] Le Gac M., Metegnier G., Chomérat N., Malestroit P., Quéré J., Bouchez O., Siano R., Destombe C., Guillou L., Chapelle A. (2016). Evolutionary processes and cellular functions underlying divergence in Alexandrium minutum. Mol. Ecol..

[21] Cho Y., Ogawa M., Yotsu-Yamashita M., Oshima Y. (2014). Effect of 5-fluoro-2′-deoxyuridine on toxin production and cell cycle regulation in marine dinoflagellate, *Alexandrium tamarense*. Harmful Algae.

[22] Wang D.-Z., Li C., Zhang Y., Wang Y.-Y., He Z.-P., Lin L., Hong H.-S. (2012). Quantitative proteomic analysis of differentially expressed proteins in the toxicity-lost mutant of *Alexandrium catenella* (Dinophyceae) in the exponential phase. J. Proteom..

[23] Zhang S.F., Zhang Y., Xie Z.X., Zhang H., Lin L., Wang D.Z. (2015). iTRAQ-based quantitative proteomic analysis of a toxigenic dinoflagellate *Alexandrium catenella* and its non-toxic mutant. Proteomics.

[24] Martins C.A., Kulis D., Franca S., Anderson D.M. (2004). The loss of PSP toxin production in a formerly toxic Alexandrium lusitanicum clone. Toxicon.

[25] John U., Litaker R.W., Montresor M., Murray S., Brosnahan M.L., Anderson D.M. (2014). Formal revision of the *Alexandrium tamarense* species complex (Dinophyceae) taxonomy: The introduction of five species with emphasis on molecular-based (rDNA) classification. Protist.

[26] Anderson D., Kulis D., Sullivan J., Hall S., Lee C. (1990). Dynamics and physiology of saxitoxin production by the dinoflagellates *Alexandrium* spp.. Mar. Biol..

[27] Benson D.A., Cavanaugh M., Clark K., Karsch-Mizrachi I., Lipman D.J., Ostell J., Sayers E.W. (2017). GenBank. Nucleic Acids Res..

[28] Hillebrand H., Dürselen C.D., Kirschtel D., Pollingher U., Zohary T. (1999). Biovolume calculation for pelagic and benthic microalgae. J. Phycol..

[29] Wang D.-Z., Gao Y., Lin L., Hong H.-S. (2013). Comparative proteomic analysis reveals proteins putatively involved in toxin biosynthesis in the marine dinoflagellate *Alexandrium catenella*. Mar. Drugs.

[30] Lee F.W.-F., Morse D., Lo S.C.-L. (2009). Identification of two plastid proteins in the dinoflagellate Alexandrium affine that are substantially down-regulated by nitrogen-depletion. J. Proteome Res..

[31] Zhuang Y., Zhang H., Hannick L., Lin S. (2015). Metatranscriptome profiling reveals versatile N-nutrient utilization, CO2 limitation, oxidative stress, and active toxin production in an *Alexandrium fundyense* bloom. Harmful Algae.

[32] Shimizu Y. (1993). Microalgal metabolites. Chem. Rev..

[33] Neilan B.A. (2014). Current knowledge of paralytic shellfish toxin biosynthesis, molecular detection and evolution. Toxins Biol. Act. Compd. Microalgae.

[34] Zhang S.-F., Zhang Y., Lin L., Wang D.-Z. (2018). iTRAQ-based quantitative proteomic analysis of a toxigenic dinoflagellate *Alexandrium catenella* at different stages of toxin biosynthesis during the cell cycle. Mar. Drugs.

[35] Maas E.W., Brooks H.J.L. (2010). Is photosynthesis a requirement for paralytic shellfish toxin production in the dinoflagellate Alexandrium minutum algal–bacterial consortium?. J. Appl. Phycol..

[36] Zhang S.-F., Chen Y., Xie Z.-X., Zhang H., Lin L., Wang D.-Z. (2019). Unraveling the molecular mechanism of the response to changing ambient phosphorus in the dinoflagellate *Alexandrium catenella* with quantitative proteomics. J. Proteom..

[37] Keller M.D., Selvin R.C., Claus W., Guillard R.R. (1987). Media for the culture of oceanic ultraphytoplankton1, 2. J. Phycol..

[38] Adachi M., Sake Y., Ishida Y. (1996). Analysis of Alexandrium (Dinophyceae) species using sequences of the 5.8 S ribosomal DNA and internal transcribed spacer regions 1. J. Phycol..

[39] Thompson J.D., Gibson T.J., Higgins D.G. (2003). Multiple Sequence Alignment Using ClustalW and ClustalX. Curr. Protoc. Bioinform..

[40] Altschul S., Gish W., Miller W., Myers E., Lipman D. (1990). Basic local alignment search tool. J. Mol. Biol..

[41] Coordinators N.R. (2016). Database resources of the National Center for Biotechnology Information. Nucleic Acids Res..

[42] Lawrence J.F., Wong B., Ménard C. (1996). Determination of decarbamoyl saxitoxin and its analogues in shellfish by prechromatographic oxidation and liquid chromatography with fluorescence detection. J. AOAC Int..

[43] Lee F.W.-F., Lo S.C.-L. (2008). The use of Trizol reagent (phenol/guanidine isothiocyanate) for producing high quality two-dimensional gel electrophoretograms (2-DE) of dinoflagellates. J. Microbiol. Methods.

[44] Wang D., Kalb S.R., Cotter R.J. (2004). Improved procedures for N-terminal sulfonation of peptides for matrix-assisted laser desorption/ionization post-source decay peptide sequencing. Rapid Commun. Mass Spectrom..

[45] Szklarczyk D., Gable A.L., Lyon D., Junge A., Wyder S., Huerta-Cepas J., Simonovic M., Doncheva N.T., Morris J.H., Bork P. (2019). STRING v11: Protein–protein association networks with increased coverage, supporting functional discovery in genome-wide experimental datasets. Nucleic Acids Res..

[46] Howell D.C. (2009). Statistical Methods for Psychology.

[47] Field A. (2013). Discovering Statistics Using IBM SPSS Statistics.

