# Supplementary material for: Production of Paralytic Shellfish Toxins (PSTs) in Toxic Alexandrium catenella is Intertwined with Photosynthesis and Energy Production
Source: Toxins (Basel). 2020 Jul 27;12(8):477. doi: 10.3390/toxins12080477 (PMC7472304; doi:10.3390/toxins12080477)
Supplement: Supplementary file 1 [file toxins-12-00477-s001.pdf]

Supplementary Materials: Production of Paralytic Shellfish Toxins (PSTs) in Toxic *Alexandrium catenella* is Intertwined with Photosynthesis and Energy Production

Sirius Pui-kam Tse, Fred Wang-fat Lee, Daniel Yun-lam Mak, Hang-kin Kong, Kenrick Kai-yuen Chan, Pak-yeung Lo and Samuel Chun-lap Lo

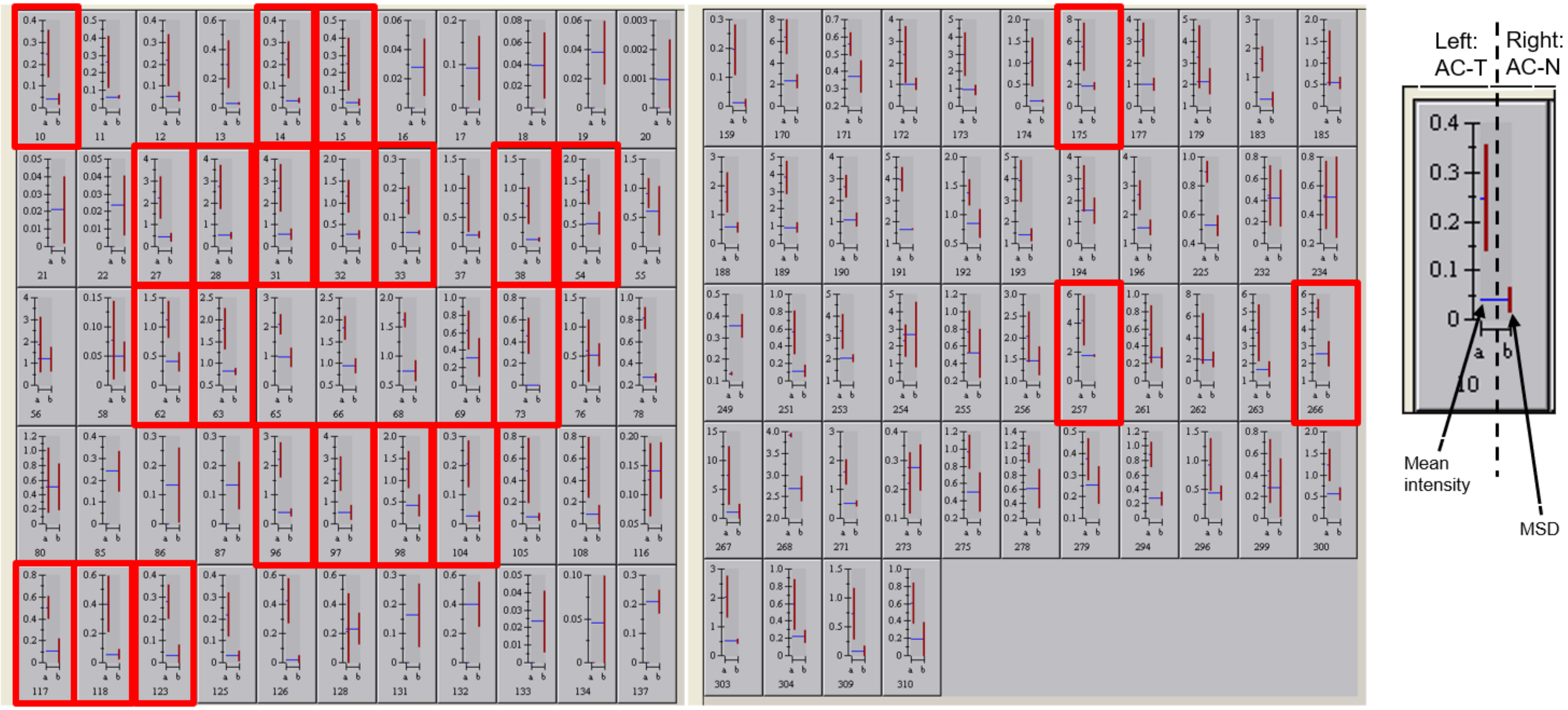

Figure S1. mean and mean square derivation (MSD) of some aligned spots found in AC-T/AC-N comparison. As described in Material and Method section, spots with mean intensity difference > 2 folds and with un-overlapped MSD bars were defined as differentially expressed protein spots and were picked for protein identification. Spots highlighted in red box indicated those spots were successfully identified, and their identities are shown in Table 1 in the Result section of the Main-text.

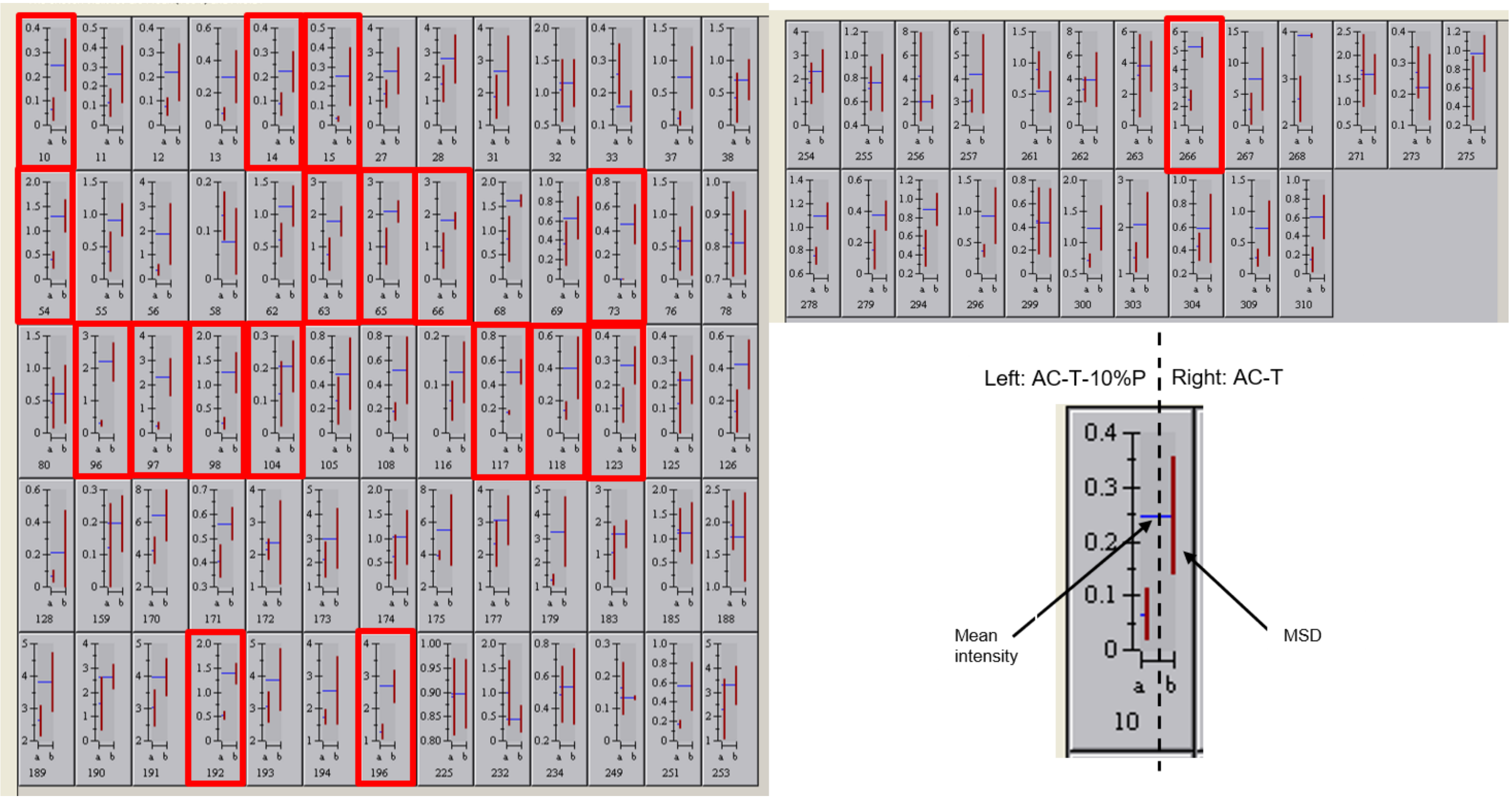

Figure S2. mean and MSD of some aligned spots found in AC-T-10%P/AC-T comparison. Spots highlighted in red box indicated those spots were successfully identified, and their identities are shown in Table 2 in the Result section of the Main-text.
